# Battery Hint

**Published:** 1879-05

**Authors:** R. Tilley

**Affiliations:** Chicago


### ARTICLE XV.

### BATTERY HINT.

No one who has used to any extent the Galvanic Battery, either for the purpose of cautery or for the general effect to be produced on the constitution, will ignore the value of any suggestion by which the constancy of the current can be improved.

The testimony of electricians on this subject is well illustrated by the fact that the relative constancy of the current of any battery is an item of great importance in the estimation of the value of said battery.

Again, any device that will dispense with the necessity of amalgamating the zincs will be, to the majority of physicians, a great source of satisfaction.

I desire to make more public a device which will greatly increase the constancy of the current, and entirely dispense with the necessity of re-amalgamating the zincs. The idea is not original with me, but in the course of conversation with several who are very familiar with the use of the battery, I have found it so little known, that I have thought it a sufficient excuse for publication. The device consists simply in the addition to the ordinary battery-fluid of a certain quantity of the bisulphate of mercury. The amount given by Gaiffe of Paris, to whom I am indebted for the idea, is four or five grams of the bisulphate of mercury to the liter of the regular sulphuric acid and bichromate fluid. Of course it is necessary to amalgamate the zincs before commencing its use. They will then remain amalgamated. I have used this for some time, and had the pleasure of recommending it to another physician in the city. The marked improvement which he has obtained in the constancy of his current and the satisfaction which he feels in not having to re-amalgamate his zincs, inspires me to affirm that any physician who once adopts this modification, will continue its use.

R. TILLEY, M.D.

CHICAGO, April, 1879.

---

MEDICAL gentlemen in this country who have contributed to the literature of the throat, voice, etc., are requested to send copies of their articles, or at least the titles, to Dr. Louis Elsberg, 614 Fifth avenue, New York.

SUBSCRIBERS of this journal will confer a great favor by sending to Rush Medical College any of its announcements of a date previous to 1850; also those for the years 1854–55, 1861–62, and for 1875–76.

